# Analysis of Compositional Gradients in Cu(In,Ga)(S,Se)_2_ Solar Cell Absorbers Using Energy Dispersive X-ray Analysis with Different Acceleration Energies

**DOI:** 10.3390/ma14112861

**Published:** 2021-05-26

**Authors:** Ulrike Künecke, Matthias Schuster, Peter Wellmann

**Affiliations:** Materials Department 6, Institute Materials for Electronics and Energy Technology, Friedrich-Alexander-University Erlangen-Nürnberg (FAU), Martensstraße 7, 91058 Erlangen, Germany; msc.matthias.schuster@fau.de (M.S.); peter.wellmann@fau.de (P.W.)

**Keywords:** Cu(In,Ga)Se_2_, solar cell, thin films, gradients, characterization, energy dispersive X-ray analysis

## Abstract

The efficiency of Cu(In,Ga)(S,Se)_2_ (CIGSSe) solar cell absorbers can be increased by the optimization of the Ga/In and S/Se gradients throughout the absorber. Analyzing such gradients is therefore an important method in tracking the effectiveness of process variations. To measure compositional gradients in CIGSSe, energy dispersive X-ray analysis (EDX) with different acceleration energies performed at both the front surface and the backside of delaminated absorbers was used. This procedure allows for the determination of compositional gradients at locations that are millimeters apart and distributed over the entire sample. The method is therefore representative for a large area and yields information about the lateral homogeneity in the millimeter range. The procedure is helpful if methods such as secondary ion-mass (SIMS), time-of-flight SIMS, or glow-discharge optical emission spectrometry (GDOES) are not available. Results of such EDX measurements are compared with GDOES, and they show good agreement. The procedure can also be used in a targeted manner to detect local changes of the gradients in inhomogeneities or points of interest in the µm range. As an example, a comparison between the compositional gradients in the regular absorber and above the laser cut separating the Mo back contact is shown.

## 1. Introduction

Photovoltaic modules consisting of CIGSSe thin-film solar cells have achieved efficiencies of 19.2% (Cd-free). This is comparable to CdTe and multicrystalline Si, but is still below crystalline Si (24.4%) [1]. Although the technology is well established, open questions remain on how to increase the efficiency to compete with crystalline Si.

One topic that has been widely discussed over the years is the optimization of the front-to-back contact Ga/In gradient of the absorber. Since the bandgap depends on the [Ga]/([Ga] + [In]) ratio (GGI), this is a way to improve solar cell performance. The control of the GGI ratio is challenging in absorbers produced by sequential processing because all selenization processes using metals or alloys as precursors normally show a Ga accumulation at the Mo back contact and CuInSe_2_ at the junction.

To judge the effect of measures taken to optimize the Ga/In gradient, simple examination procedures are needed. The gradient is directly accessible on the cross section in a front-to-back EDX line scan. Cross sections can be produced by breaking the sample or by a combination of mechanical polishing and Ar beams [2]. Breaking always leads to an uneven surface with fracture planes along the grain boundaries. This causes an additional uncertainty in EDX analysis because of the increased absorption of lower energy X-rays in depressions. This problem can be avoided by the second, but more complex, method. Common to both preparation methods is that a high spatial resolution is mandatory since the line scan has to be performed on a layer of below 2 µm thickness that is immediately adjacent to the Mo back contact and the glass substrate. High spatial resolution can be achieved by using acceleration energies below 10 keV [3]. This approach gives exact results but does so only for one location, which may not necessarily be representative for the whole absorber. Because of the low X-ray yield, it also places high demands on signal detection requiring a high-resolution detector.

SIMS, GDOES, and others (see overview in [4]) offer a way to measure the compositional gradients from the surface of the absorber on a statistically relevant area. Concerning the ability to detect trace elements, SIMS and GDOES surpass the sensitivity of EDX by far. They can also measure the Na content, which is of great interest in CIGSSe solar cells. This is problematic in an EDX integrated in a scanning electron microscope (SEM) because of the overlap of the Mo and Na lines and the low Na concentration in the samples. SIMS and GDOES have a depth resolution in the nm scale under ideal measurement conditions. In EDX, the depth resolution is determined by the size of the X-ray generation volume, which depends on the energy of the beam electrons and the material. It can range between a few 100 nm and several µm. SIMS, GDOES, and others are very sensitive tools and offer many advantages over EDX but have the disadvantage of being a less common laboratory equipment than a SEM equipped with EDX.

The motivation for this work is to use standard SEM/EDX equipment to determine the gradients of the main constituents in a statistically relevant way. This makes it possible to compare the gradients in absorbers from different process variations. EDX line scans over the layer cross section are difficult to compare because the absorbers show lateral fluctuations in layer thickness, composition, and contain pores. Instead of examining the cross section, the analysis is carried out on the surface and the backside of the delaminated absorber. The acceleration energy is varied, taking advantage of the fact that the depth of X-ray generation depends on the acceleration energy. Such measurements can be performed at many random locations and averaged if the aim is to examine differences between process variations. Another application is the characterization of inhomogeneities. Gradients in local inhomogeneities in the µm range can be determined and compared to those in the regular absorber.

## 2. Materials and Methods

### 2.1. Absorber Production

The CIGSe absorbers were produced at the Avancis GmbH pilot line (Munich, Germany) by the stacked elemental layer–rapid thermal process [5,6] on soda lime glass substrates covered by a SiN alkali-barrier layer. The Mo back contact contained an intermediate selenization barrier. The precursor layer consisted of DC magnetron sputtered Cu–In–Ga upon which a Se layer was thermally evaporated. Rapid thermal processing (RTP) was conducted in an infrared heated furnace capable of high heating rates with or without a S containing gas. EDX with different acceleration energies was performed on two different types of samples.

**Figure 1:**
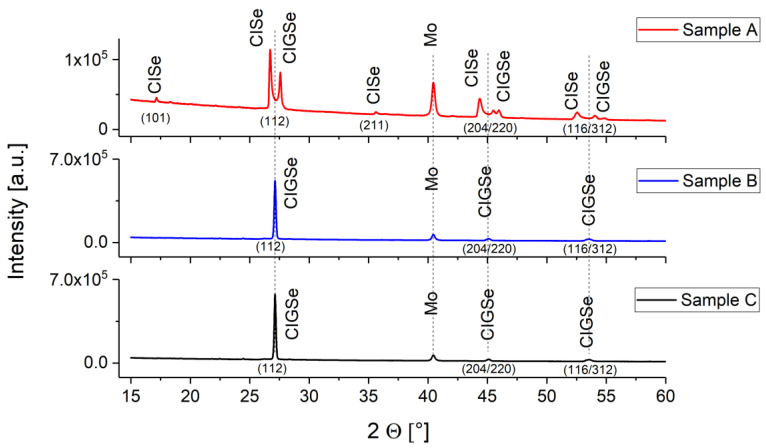
X-ray diffraction measurements on samples A, B, and C.

Samples from three different process variations were used to demonstrate how this method can be applied to determine differences in the compositional gradients. These samples contained no S. During growth, the Se supply was decreased in the following order: sample A > sample B > sample C. The lower Se activity was achieved by reducing the Se amount in the precursor and by removing Se from the reaction atmosphere during the RTP. No buffer and i-layer were deposited. X-ray diffraction (XRD) measurements in Bragg Brentano configuration show that sample A is a two phase sample consisting of In-rich and Ga-rich Cu(In,Ga)Se_2_ while samples B and C are single phase and show mixed Cu(In_x_Ga_1−x_)Se_2_ (Figure 1). None of the phases that occur as precursors before and during the formation of CIGSe could be detected.

**Figure 2:**
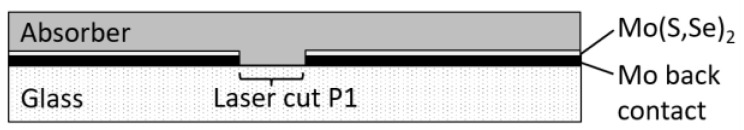
P1 laser cut.

To show how EDX with different acceleration energies can be used to detect local changes in the compositional gradients in small distinctive regions such as the area above the P1 laser cut (which is a few 10 µm wide), measurements were performed on an additional S containing sample D. The P1 laser cut separates the Mo back contact. The laser patterning was performed before the deposition of the precursor layer. Figure 2 shows the P1 laser cut schematically. No buffer and i-layer were deposited. Table 1 gives an overview over the samples used.

### 2.2. Characterization Methods

The composition was determined in a FESEM Jeol JSM-7610F (Jeol LTD, Akishima, Tokyo, Japan) with an Oxford EDX system equipped with an 80 mm^2^ X-Max silicon drift detector using AZtec 3.1 (Oxford Instruments PLC, Abingdon, UK), standardless mode with XPP-correction. The probe current was set to 0.45 nA. Between 15 to 30 keV, the Cu(K), Ga(K), In(L) and Se(L) lines were used for analysis. The choice of lines is discussed in Section 2.4. The absolute accuracy of standardless EDX measurements is 2 to 20% [7].

The compositional gradients in samples A, B, and C were determined with EDX in several ways: (i) on the cross section by EDX line scans from the front to the back of the absorber (perpendicular to the back contact) at 20 keV; (ii) on the cross section by several EDX line scans parallel to the back contact, taken at different distances from the back contact at 20 keV; and (iii) on the surface and the backside of the absorber across an area of 25 × 25 µm^2^ using different acceleration energies (15, 20, 25, and 30 keV) at 12 different positions on the sample. To examine the compositional changes above the P1 laser line, the measurement was performed above P1 and on the regular absorber as described in (iii) but on a smaller area of 20 × 20 µm^2^. The backside of the absorber was made accessible by delaminating it from the back contact. The separation occurs reproducibly between the MoSe_2_ and the absorber.

The cross sections were prepared by breaking the sample. Sample drift was automatically corrected for the measurements on the cross section. All samples were coated with carbon to prevent charging.

GDOES was measured at the Helmholtz Zentrum Berlin by order of Avancis using a Spectrum Analytik GDA650 system (GmbH, Hof, Germany) in pulsed radio frequency (rf) mode on samples from the same production run. Details on the measurement, sputter parameters, and quantification procedure can be found in [8].

### 2.3. X-ray Generation Volume

**Figure 3:**
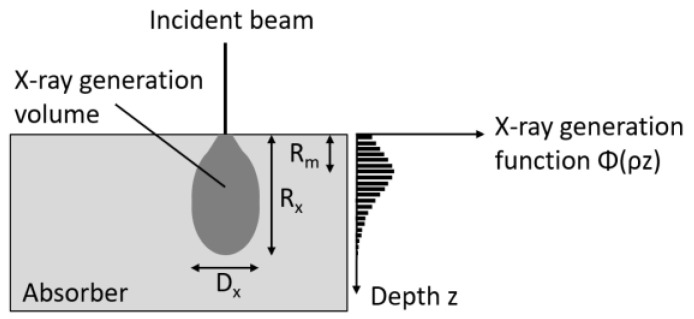
Schematic representation of X-ray range R_x_, R_m_, and D_x_.

EDX measurements give the average composition inside a spherical volume from which the X-rays are generated by inelastic scattering of the beam electrons. The size of the X-ray generation volume can be characterized by the X-ray range R_x_ and the lateral diameter of interaction D_x_. D_x_ is the projection of the maximum width of the X-ray generation volume to the surface and is almost equal to the spatial resolution [9]. R_x_ is the depth in the sample where no X-ray generation is possible any more (Figure 3). R_x_ can be calculated after the general Kayama-Okayama expression, where E_0_ is the energy of the incident beam, E_c_ the critical energy necessary for the excitation of the respective X-ray line, and ρ the density of the matrix.

(1) $$R_{x}=K(E_{0}{}^{n}-E_{c}{}^{n})\frac{1}{\rho }$$

According to Willich and Bethke, D_x_ can be assumed to be similar to R_x_ [10]. There are other estimations for D_x_ giving larger results [11] or considering the dependence on Z [3]. Here, the approximation of Willich and Bethke is used.

Different values for K and n in the Kayama-Okayama expression have been proposed in literature and are quoted in Table 2. Only Castaing [12] used a composition dependent K factor. It was calculated for CuIn_(1−x)_Ga_x_Se_2_ with x = 0.25 using an averaged atomic weight A and atomic number Z for the compound. As can be seen, the K factors differ only slightly for the different methods.

The X-ray range R_x_ was calculated after the different methods for all lines in CIGSe. The Castaing method results in bigger values than that of Anderson and Hasler, and the values calculated after Reed are the smallest. Therefore, the method of Anderson and Hasler was chosen for the calculation of the X-ray range.

The distribution of the X-ray production is not uniform laterally and in depth inside the spherical volume defined by D_x_ and R_x_. Monte Carlo simulations of the electron trajectory paths in the sample show that the X-ray production first increases with distance from the surface and then reaches a maximum at the depth R_m_ from which it declines until R_x_ is reached, where no ionization is possible any more [9]. R_x_, R_m_, and D_x_ are visualized in Figure 3.

Since most of the ionization occurs around R_m_ and not at R_x_, R_m_ is used as a characteristic value for the depth at which the X-rays are generated. R_m_ can be calculated according to [3] after:

(2) $$R_{m}=\frac{1}{3}R_{x}$$

### 2.4. Choice of X-ray Lines

Since the measurements are conducted at acceleration energies ranging from 15 to 30 keV, the lines have to be chosen in a way that all of them are excited close to the optimum while keeping the problem of reabsorption and fluorescence as small as possible. Our own experiments showed that it is preferable to use the same lines throughout the energy variation since the quantification results slightly depend on the choice of lines.

Excitation of a specific line is only possible if the overvoltage, the quotient of the acceleration energy, and the critical energy of excitation, is bigger than one. Since the cross section of inner shell ionization has a maximum at an overvoltage of ca. 3 and decreases for higher values, an overvoltage of 2–3 is usually considered as optimum [9]. All low energy lines (L-lines for Cu, Ga and Se, M-line for In) show a very high overvoltage while Se(K) is not sufficiently excited at 15 keV.

Since the X-rays are produced below the surface of the sample, they undergo absorption while travelling to the surface. The attenuation lengths (drop to 1/e) for the respective lines were compared to R_m_ for the take-off angle of 35° of the EDX detector. For R_m_ bigger than the attenuation length, absorption plays a role, even though it should be corrected by the EDX software. Table 3 gives the calculated values for R_m_ according to Equations (1) and (2) using K and n according to Anderson and Hasler and the attenuation length taken from [14].

Shown shaded in gray are the conditions under which R_m_ is larger than the attenuation length. Therefore, for the energy variation, the Cu(K), Ga(K), and In(L) lines were chosen. Se(L) was chosen over Se(K) despite its small attenuation length because the latter is not sufficiently excited at 15 keV. This choice is supported by the fact that the Oxford software automatically uses the Se(L) line in the range between 15 and 30 keV.

The average R_m_ for the chosen lines is 0.3 µm at 15 keV, 0.5 µm at 20 keV, 0.8 µm at 25 keV, and 1.1 µm at 30 keV.

The only lines where fluorescence caused by reabsorption could play a role are Se(L) and In(L) because the attenuation length for Cu(K) and Ga(K) is even larger than R_x_. Since fluorescence in practice only happens if the energy of the exciting X-rays is ca. 3 keV bigger than the critical energy [9], the Se(L) and In(L) radiation are not probable to cause fluorescence in CIGSe. Of course, fluorescence by bremsstrahlung is possible for all elements in the compound. Matrix effects like absorption and fluorescence are corrected by the EDX software. We consider the effect of elemental gradients on this correction as negligible.

## 3. Results

### 3.1. Experimental Evaluation of the X-ray Generation Volume

For the measurements on the cross section, the X-ray generation volume extends into the absorber parallel to the back contact. Since the Ga/In content changes perpendicular to the back contact, the lateral diameter of interaction D_x_ is of greater interest than the X-ray range R_x_ and R_m_. At 20 keV, R_x_ and D_x_ are 1.5 µm. D_x_ and R_x_ define the outer limits of the generation volume while most of the radiation comes from inside the generation volume. Because of that, the lateral diameter of interaction was experimentally determined by using the increase of the Si signal from the glass substrate. At 20 keV, the experimentally determined D_x_ is ca. 0.9 to 1 µm. Since the absorbers are only 1.6 µm thick, this is still large. Therefore, the elements contained in the back contact as well as in the glass substrate were deconvoluted in all line scans.

The lateral resolution can be significantly improved by using beam energies below 10 keV and the low energy (L)-lines [3]. Still 20 keV was chosen since in this experiment the line scans should serve for comparison with the data from the energy variation.

For the EDX measurements with different acceleration energies performed with the beam perpendicular to the surface and back of the delaminated absorber, the situation is different because the Ga/In content changes in the same direction. The lateral diameter of interaction D_x_ is irrelevant since the measurement is conducted over an area of 25 × 25 µm^2^ anyway and is meant to average over the lateral deviations in composition. The generation depth R_x_ and the depth of maximum ionization R_m_ become the important variables since they determine how far the measurement extends into the absorber. At 15 and 20 keV, both the average R_m_ and the average R_x_ are inside the absorber layer. Hence, no major distribution from the back contact or glass should be expected. At 25 and 30 keV, the average positions of maximum radiation are 0.8 and 1.1 µm, respectively, and therefore still inside the 1.6 µm absorber layer. The average R_x_ is 2.4 and 3.3 µm, indicating that part of the generation volume extends into the back contact and glass. To see the extent to which signals from the back contact and glass are present, it was experimentally determined on sample C when the Mo (L) and Si (K) peaks begin to show up. For Mo, this is the case at 20 keV with a content calculated to 0.2 at%, reaching 1.8 at% at 30 keV. Si starts to show up at 23 keV with 0.3 at% but never exceeds 0.6 at%.

To exclude the small influence of elements contained in the back contact or glass substrate on the quantitative analysis, they were deconvoluted in the EDX measurements. It can be assumed that the elements contained in the back contact and glass do not distort the results of the EDX measurements at 25 and 30 keV and that the use of R_m_ is closer to reality than that of R_x_. For the measurements from the backside, influences of the back contact and glass are impossible since both are not present any more.

### 3.2. EDX Line Scans Perpendicular to the Back Contact

**Figure 4:**
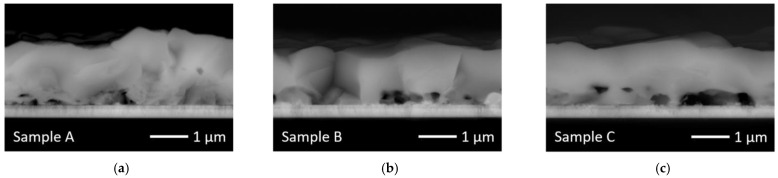
Typical appearance of absorber cross sections: (**a**) sample A; (**b**) sample B; (**c**) sample C.

Figure 4 gives the typical appearance of the absorber layers in a cross section prepared by breaking. All three absorbers exhibit an average thickness of ca. 1.6 µm with a thickness varying between 1.2 and 2 µm. Even though several line scans were performed for each sample, this variation in thickness does not allow for averaging the line scans. This is a major disadvantage because a single line scan can hardly be representative for the whole sample, especially since lateral deviations in composition and pores above the back contact are present to some degree. Above the pores, the remaining absorber thickness is substantially reduced, resulting in a locally changed Ga/In gradient.

**Figure 5:**
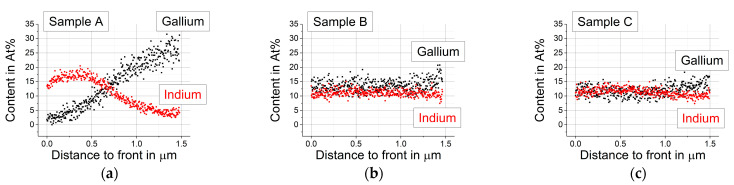
EDX line scans performed on the cross section at 20 keV: (**a**) sample A; (**b**) sample B; (**c**) sample C.

Another problem is that a flat fracture surface, as required for an ideal sample for EDX, is the exception and not the rule because different grains break differently. The low energy Se(L) signal is especially influenced by the uneven surface. Depressions emit a reduced Se (L) signal because of reabsorption at the edges while the higher energy Cu(K) and Ga(K) signals are not as much affected. Because of this, no [Ga]/([In]+[Ga]) ratios were calculated from the line scans. Nevertheless, the EDX line scans shown in Figure 5 clearly indicate that the Ga gradient is strong in sample A with high Se supply, and the Ga content is almost homogeneous for samples B and C with a reduced Se supply.

### 3.3. EDX Line Scans Parallel to the Back Contact

**Figure 6:**
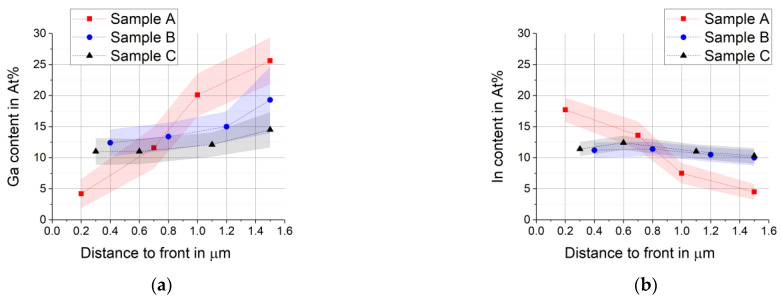
EDX line scans at 20 keV on the cross section parallel to the back contact. Points represent average of the line scan at the distance given, shaded area represents the standard deviation, the connecting lines between individual points serve as guide for the eye: (**a**) Gallium gradient; (**b**) Indium gradient.

To overcome the disadvantages of line scans perpendicular to the back contact mentioned in Section 3.2, additional line scans that were ca. 6 µm long were taken parallel to the back contact at four different distances. The average composition and standard deviation (shown as shaded area) were calculated for each line scan and plotted against the distance of the line scan to the front of the absorber. The results for Ga and In are given in Figure 6.

The more lateral inhomogeneities are present along the line scan, the larger the standard deviation will be. Close to the back contact, where pores are present, the Se, Ga, and Cu standard deviations are therefore increased.

Even though this type of measurement introduces averaging, it still depends on where on the sample the operator chooses to perform the line scan. Long range inhomogeneities are not detectable. Furthermore, the result of the measurement is influenced by the number of pores present and the flatness of the surface of the cross section. Since the width of the line scan is given by D_x_, which is 0.9 to 1 µm, the line scans on the 1.6 µm thick absorber layer overlap strongly.

**Figure 7:**
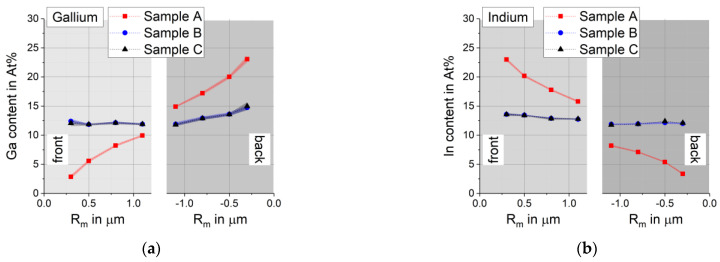
EDX with different acceleration energies measured from the front and the back. Points give the average between 12 different locations and shaded area represents the standard deviation: (**a**) Ga gradient; (**b**) In gradient.

Again, it is clearly visible from Figure 6 that sample A with the highest Se supply shows the strongest In and Ga gradients. The difference between the Ga content at the front and at the back is the largest for A, followed by B, and small for C with the lowest Se supply. The difference between samples B and C is most likely just a local coincidence since it cannot be seen in the line scans taken perpendicular to the back contact (Figure 5) or in the EDX measurements with different acceleration energies taken at the front and the back of the delaminated absorbers (Figure 7).

### 3.4. EDX Measurements with Different Acceleration Energies from the Front and the Back

EDX measurements over 25 × 25 µm^2^ were conducted at 12 different locations on both sides of each sample at energies ranging from 15 to 30 keV. The average composition and standard deviation were calculated for each energy. In Figure 7 they are shown plotted against the average depth of maximum excitation R_m_ for this energy. The connecting lines between individual points serve as guide for the eye only. The average R_m_ for the chosen lines is 0.3 µm at 15 keV, 0.5 µm at 20 keV, 0.8 µm at 25 keV, and 1.1 µm at 30 keV (see Table 3). Positive values of R_m_ indicate that the measurement was performed from the front side, while negative values of R_m_ indicate that the measurement was performed from the back side. For the same absolute value R_m_ at the front and back, the location of the X-ray production volume in the sample is different.

The EDX measurements performed with different acceleration energies exhibit a very small standard deviation between different locations, indicating that the long-range homogeneity is very good. Samples B and C show no significant differences in their Ga and In profiles, even though they were processed with different Se supplies. Only the initial reduction of the Se supply from sample A to B has a great impact on the Ga/In gradients. Sample A shows the well-known strong enrichment of Ga close to the back contact corresponding with an In-depletion, while samples B and C exhibit an almost homogeneous Ga and In content.

**Figure 8:**
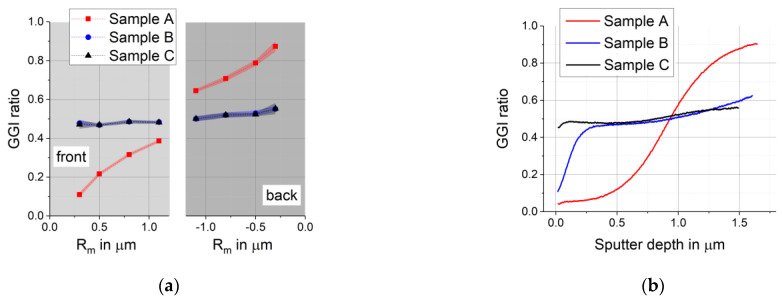
[Ga]/([Ga]+[In]) ratio (GGI ratio) calculated from: (**a**) EDX measurements with different acceleration energies at the front and back of the delaminated absorber; (**b**) GDOES measurements.

From the results shown in Figure 7, the [Ga]/([In] + [Ga]) ratios were calculated and plotted against the average maximum of X-ray production R_m_. In Figure 8 they are compared to GDOES measurements on samples from the same production run. The depth resolution of GDOES measurements is determined by the curvature of the bottom of the sputtered crater and sputter induced roughening of the samples [8] and is typically 5 to 10% of the layer thickness [4].

The connecting lines between individual [Ga]/([In] + [Ga]) ratios calculated from EDX with different acceleration energies serve as guide for the eye only. EDX shows the same trend as GDOES does for all samples with the exception of sample B close to the surface. Sample A has a steep Ga/In gradient with a very low Ga content at the front and strong Ga enrichment at the back (see Table 4 and Table 5). Sample C with the lowest Se supply has a high GGI close to the front which rises slightly and almost linearly to the back. The same applies for sample B outside a 300 nm wide surface region where a reduced GGI is seen only in GDOES. This was further analyzed with additional EDX measurements at 10 kV and is discussed below. Figure 8 shows that both methods depict the progression of the GGI between surface and backside very similarly for sample C and sample B away from the surface. Sample A with its strong Ga/In gradient shows a different course of the curve in GDOES and EDX. GDOES with its better depth resolution measured an S-shaped increase of the GGI with a convex curvature in the upper part and a concave curvature in the lower part of the absorber and an inflection point in between. EDX with different acceleration energies is not able to reproduce this S shape because it measures the average composition inside a volume that increases with increasing depth.

Table 4 and Table 5 give the [Ga]/([In] + [Ga]) ratios close to the front and back calculated from GDOES, the averaged line scan at 20 keV, area EDX at 15 keV, and additional area EDX measurements at 10 keV using Ga and Cu L-lines. The agreement between different types of measurement is very good at the back for all samples (Table 5). The reduced GGI at the front seen for sample B in GDOES cannot be seen in any of the EDX measurements, not even in those at 10 kV (Table 4). EDX measures the Ga content in a volume close to the surface defined by R_m_ and R_x_. At 10 keV, the average R_m_ is 0.2 and R_x_ is 0.5 µm. The maximum of the X-ray production lies inside the 300 nm region but part of the excitation happens below 300 nm. At least a reduction of the GGI should therefore be expected to be seen. GDOES shows a better depth resolution of 5 to 10% of the absorber thickness than EDX, but very close to the surface GDOES can also deliver inaccurate values because the sputter rate is not constant yet. This effect can be seen for all samples in Figure 8b very close to the surface, but it should not continue 300 nm deep into the absorber. It should also be noted that GDOES and EDX were performed on different samples from the same production run and that the irregularity causing the differences in the front region of sample B might not be present in the samples used in EDX.

### 3.5. EDX Measurements with Different Acceleration Energies Applied on Inhomogeneities in the µm Range

**Figure 9:**
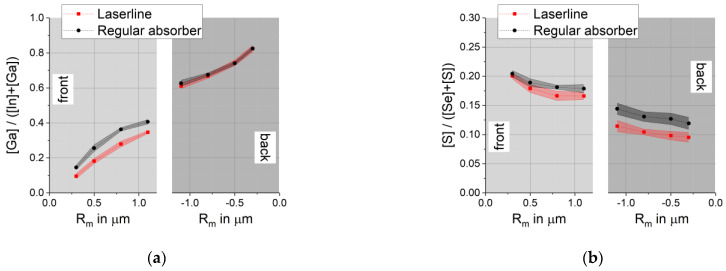
Comparison between the regular absorber and the absorber above the laser cut for sample D: (**a**) [Ga]/([In] + [Ga]) ratio calculated from EDX with different acceleration energies; (**b**) [S]/([Se] + [S]) ratio calculated from EDX with different acceleration energies.

EDX measurements with different acceleration energies can also be used to characterize inhomogeneities in the µm range, as long as they are visible from the front and the back side of the delaminated absorber. As an example, the region above the a few ten µm wide P1 laser line of the S containing sample D was examined. Compared to the regular absorber, the absorber in this region is ca. 0.3 µm thicker and exhibits a smoother surface at the front and an increased porosity at the back. EDX measurements with different acceleration energies as described above were performed and the [Ga]/([In] + [Ga]) and [S]/([Se] + [S]) ratios calculated. They are given in Figure 9 as a function of the average depth of maximum excitation R_m_ for the respective energy. The connecting lines between individual ratios serve as guide for the eye only.

EDX with different acceleration energies showed differences between the laser line and the regular absorber that are responsible for the changes in morphology visible on the front and the back of the absorber. The absorber above the laser line contained less Ga close to the front while the [S]/([Se] + [S]) ratio was reduced throughout the absorber. GDOES or SIMS are not able to detect local changes of compositional gradients on such a small scale.

## 4. Discussion

The differences between the three samples with a variation of the Se supply can be detected by all three methods, i.e., line scans with and without averaging as well as EDX with different acceleration energies. Even though the last method cannot provide absolute values of the composition since the size and shape of the analyzed volume change during the measurements, it has several advantages over the traditional line scan across the absorber layer.

Many different locations on the sample can be analyzed, and the results can be averaged. By this, one can gain statistically relevant information on the compositional gradients, as well as on the homogeneity in the mm range and obtain a result that is representative for a large area. This allows us to compare the compositional gradients between process variations. For the traditional line scan at the cross section, an averaging of line scans taken at different locations is not possible because the thickness of the absorber is usually not the same.

EDX measurements with different acceleration energies do not require a special sample preparation as is necessary to achieve a flat surface of the cross section for a high-quality line scan. Even if the surface is perfectly flat, the results of a line scan are influenced by the reabsorption of the low energy lines if pores are present. This type of absorption is not corrected by the EDX software since the software assumes a perfectly flat surface.

EDX measurements taken with different acceleration energies can also be used to compare the compositional gradients between regions of interest visible only in the SEM, as was shown here exemplarily for an absorber close to the laser line and away from it. Performing traditional line scans on such regions of interest becomes rather challenging if the region of interest is small and detectable only in SEM. Since it is impossible with SIMS and GDOES, this is a unique advantage of the method although it is restricted to the main constituents.

EDX measurements taken with different acceleration energies allow users to choose the lines for the analysis according to the capability of their SEM and EDX equipment and software. For EDX line scans with a small spatial resolution, one is forced to use acceleration energies below 10 keV. This leads to a restriction on the low energy (L)-lines that lie close together and have a low X-ray yield requiring a high-resolution detector and a sufficiently high beam current. The measurement becomes very surface sensitive so that thin surface layers can be detected. This implies that contamination, coatings, and the roughness of the surface need to be considered. In addition, sample drift becomes problematic and needs to be corrected accordingly.

## Figures and Tables

**Table 1:** Sample overview.

Sample	Se Supply	S Supply	P1 Processed
A	Regular	None	No
B	<Regular	None	No
C	<<Regular	None	No
D	Regular	Regular	Yes

**Table 2:** Different K and n factors for the calculation of the X-ray range R_x_.

Method	K	*n*
Anderson and Hasler [13]	0.064	1.68
Reed [11]	0.077	1.5
Castaing [12]	0.033 × A/Z = 0.070 for CuIn_0.75_Ga_0.25_Se_2_	1.7

**Table 3:** Depth of maximum ionization R_m_ and attenuation length for different X-ray lines in CIGSe.

Atten. Length, R_m_	Cu (K)	Cu (L)	Ga (K)	Ga (L)	Se (K)	Se (L)	In (L)	In (M)
Att. length [µm]	8.12	0.21	7.83	0.21	13.10	0.35	1.46	0.08
15 keV, R_m_ [µm]	0.23	0.35	0.20	0.35	0.14	0.35	0.33	0.35
20 keV, R_m_ [µm]	0.45	0.57	0.42	0.57	0.36	0.57	0.55	0.57
25 keV, R_m_ [µm]	0.71	0.83	0.68	0.83	0.62	0.83	0.81	0.83
30 keV, R_m_ [µm]	1.01	1.13	0.98	1.13	0.92	1.13	1.10	1.13

**Table 4:** [Ga]/([In] + [Ga]) ratios close to the front of the absorber calculated from different types of measurement.

Type of Measurement	Sample A	Sample B	Sample C
GDOES	0.05	0.10	0.45
EDX, averaged line scan, 20 keV	0.19	0.52	0.49
EDX, area, 15 keV	0.11	0.48	0.47
EDX, area, 10 keV	0.07	0.52	0.52

**Table 5:** [Ga]/([In] + [Ga]) ratios close to the back of the absorber calculated from different types of measurement.

Type of Measurement	Sample A	Sample B	Sample C
GDOES	0.90	0.63	0.56
EDX, averaged line scan, 20 keV	0.85	0.66	0.59
EDX, area, 15 keV	0.87	0.55	0.55
EDX, area, 10 keV	0.89	0.61	0.56

## Data Availability

Data is contained within the article.
